# Zika mosquito vectors: the jury is still out

**DOI:** 10.12688/f1000research.9839.1

**Published:** 2016-10-20

**Authors:** Walter S. Leal

**Affiliations:** 1 Department of Molecular and Cellular Biology, University of California-Davis, Davis, USA

**Keywords:** Zika, Aedes aegypti, Culex quinquefasciatus, Chikungunya, microcephaly, Guillain-Barré syndrome

## Abstract

After a 40-year hiatus, the International Congress of Entomology (ICE 2016) convened in Orlando, Florida (September 25-30, 2016). One of the symposia at ICE 2016, the Zika Symposium, covered multiple aspects of the Zika epidemic, including epidemiology, sexual transmission, genetic tools for reducing transmission, and particularly vector competence. While there was a consensus among participants that the yellow fever mosquito, *Aedes aegypti*, is a vector of the Zika virus, there is growing evidence indicating that the range of mosquito vectors might be wider than anticipated. In particular, three independent groups from Canada, China, and Brazil presented and discussed laboratory and field data strongly suggesting that the southern house mosquito, *Culex quinquefasciatus*, also known as the common mosquito, is highly likely to be a vector in certain environments.

## Background

The International Congresses of Entomology (ICEs) are the “Olympics of Entomology,” which started in 1910 in Brussels, Belgium. It is only the third time that ICE has been held in the United States of America (USA), the last conference being 40 years ago when delegates convened in Washington, DC for XV ICE. This time delegates from 102 countries convened in Orlando, Florida (September 25–30, 2016) for a historic event titled “Entomology Without Borders.” With 6,682 delegates, ICE 2016 was undoubtedly the largest gathering of scientists in the history of entomology.

**Figure 1.**
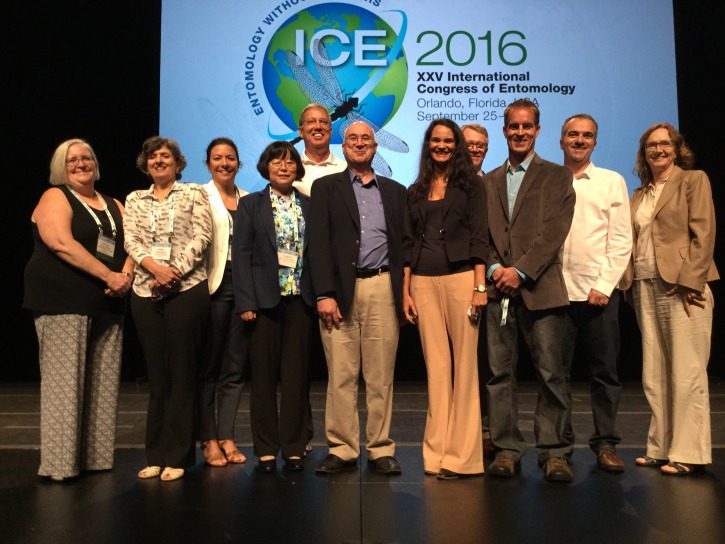
Snapshot of the Zika Symposium at the 2016 International Congress of Entomology in Orlando, FL. From the left: Dr. Adriana Costero; Dr. Celina Martelli; Dr. Duschinka Guedes (back row); Dr. Tang-yan Zhao; Dr. Anthony A. James (back row); Dr. Stephen Higgs; Dr. Constância Ayres; the author (back row); Dr. Brian D. Foy; Dr. Luciano Moreira; and Dr. Fiona F. Hunter.

This Opinion article is based mainly on the current literature and the Zika Symposium at ICE 2016, which was organized by Dr. Constância Ayres, Oswaldo Cruz Foundation (FIOCRUZ-PE), Recife, Brazil, and Dr. Adriana Costero, National Institutes of Health, Bethesda, Maryland. The symposium featured the following speakers ( Figure 1): Dr. Celina Martelli, Centro de Pesquisas Aggeu Magalhães, FICRUZ-PE, Brazil; Dr. Stephen Higgs, Kansas State University, Manhattan, Kansas; Dr. Brian D. Foy, Colorado State University, Fort Collins, Colorado; Dr. Constância Ayres, FICRUZ PE, Dr. Duschinka Guedes, FIOCRUZ-PE; Dr. Luciano Moreira, FIOCRUZ-MG; Dr. Anthony A. James, University of California, Irvine; Dr. Fiona F. Hunter, Brock University, Canada; and Dr. Tang-yan Zhao, Institute of Microbiology and Epidemiology, Beijing, China; and was attended by hundreds of delegates.

### Zika history

At the time we were preparing to submit a bid on behalf of the Entomological Society of America to host ICE 2016 in Orlando and during the first years of preparation for the conference, Zika was not part of the vocabulary of a large majority of entomologists, myself included. The Zika virus (ZIKV) was isolated first from a febrile monkey and later from the mosquito *Aedes africanus* about the time our predecessors were preparing to host VIII ICE in Stockholm ^1^. After Sweden in 1948, ICE convened in Amsterdam, Montreal, Vienna, London, Moscow, Canberra, Washington, Kyoto, Hamburg, Vancouver, Beijing, Florence, Iguaçu Falls, Brisbane, Durban, and Daegu (South Korea), and yet ZIKV was not part of our jargon. During these years while ICE delegates were travelling throughout the world to advance the field of entomology, ZIKV was silently making headway out of the Zika forest (Entebbe, Uganda), hitchhiking on humans, and conquering new habitats. As it reached new environments, the virus was likely being transmitted locally by native species of mosquitoes and/or other “illegal immigrants” as, for example, the notorious yellow fever mosquito, *Aedes aegypti.* ZIKV was isolated from humans for the first time in 1954 during an outbreak of jaundice suspected of being yellow fever. The virus was isolated from one of the three patients examined, and the other two exhibited high titers of serum antibodies against the virus ^2^. As Dr. Stephen Higgs pointed out in his presentation, outbreaks occurred in Asia between 1954 and 2007, and up to that point when the virus reached Yap Island only 14 human cases had been identified, so no one paid much attention to the virus. However, on Yap Island, Federal State of Micronesia, it was estimated that 73% of residents 3 years of age or older have been infected with ZIKV ^3^, which is characterized by rash, conjunctivitis, and arthralgia. In an attempt to identify the mosquito vector, they collected adults from the field and found that *Aedes hensilli* and *Culex quinquefasciatus*, 41.2 and 28.1%, respectively, were the predominant species. Because the virus was not found in any field-collected *Aedes* mosquitoes, they conducted laboratory studies to determine the vector competence of *Ae. hensilli* to transmit ZIKV. About 80% of the mosquitoes fed through the Hemotek ^®^ feeding system on ZIKV-containing sheep blood became infected, but only 13–23% developed dissemination infections. By contrast, 60% of the mosquitoes fed blood-containing Chikungunya virus (CHIKV) became infected and 80% disseminated. Their findings supported the possibility that *Ae. hensilli* served as a vector during the Zika outbreak on Yap Island.

### Preparing for Chikungunya and Zika major outbreaks

Up until 2013, ZIKV infection was considered a mild infection, but in French Polynesia the symptoms were complicated by Guillain-Barré syndrome ^4^, a rare neurological disorder identified 100 years ago. It has been estimated that between September 2013 and March 2014 as many as 28,000 patients were affected, i.e., ca. 11% of the Polynesian population ^5^. The next and most notorious ZIKV outbreak occurred in Brazil, the locale from which the virus has been moving rapidly up toward North America and back to Africa. Apparently, ZIKV went under the “radar screen” soon after the arrival of CHIKV. Brazil was expecting an outbreak of CHIKV; primary care physicians (PCPs) had been trained at least two years in advance on how to distinguish CHIKV symptoms from those elicited by the Dengue virus (DENV). The first local transmission of CHIKV in Brazil was reported on September 16, 2014 ^6^. Epidemiologists and PCPs created a network using WhatsApp, “Chickv – the mission,” to share information and get better prepared for the epidemic. While working on CHIKV, they started observing cases sometimes referred to as “dengue fraca” (weak dengue), which was characterized by low fever and an intense allergic reaction. The detective work of medical doctors prompted epidemiologists to consider the possibility of another virus outbreak. ZIKV was detected in Brazil for the first time in April/May 2015. According to an investigative account by professor of bioethics, Debora Diniz ^6^, the first publication on the occurrence of ZIKV in Brazil ^7^ was authored by the second group to isolate the virus. The first group to report their findings to the media (on April 29, 2015) published their findings in a peer-reviewed journal ^8^ five months after the first scientific report ^7^.

### Microcephaly

During the Zika Symposium, Dr. Celina Martelli reported that in August–September 2015, neurologists, neonatologists, and epidemiologists became more aware of neonates with microcephaly, and in October clinical data and brain imaging suggested a congenital infection. Then, they noticed an important correlation between the major circulation of ZIKV in the Northeast region of Brazil and the time of the first gestational months of mothers. Thereafter, ZIKV was detected in amniotic fluid ^9^, and it is now well established that ZIKV causes microcephaly ^10^. It was this teratogenic effect, unique for a flavivirus ^11^ and arboviruses in general, coupled with its rapid dissemination in Latin America that led the World Health Organization on February 1, 2016 to declare ZIKV an international public health emergency.

### Sexual transmission

ZIKV is the only arbovirus currently known to also be sexually transmitted. Speaking at ICE 2016, Dr. Brian Foy suggested that sexual transmission is underestimated in epidemiological data. He pointed out that there are two main causes for this underestimation. It is difficult to decouple sexual from mosquito-borne transmission in outbreak areas and questionnaires regarding patients’ sex life tend to be inaccurate for various reasons. The case study of Dr. Foy and his wife in the United States was the first report to suggest sexual transmission of ZIKV. Five to six days after returning from field research in Senegal, Dr. Foy had typical symptoms of arbovirus infection, and a few days later his wife started developing similar symptoms, although her headaches and photophobia were more intense and arthralgia remained for months. There were no infected mosquito vectors in the area, their children did not get sick, and Dr. Foy had no reason to “lie on a questionnaire.” He immediately suspected sexual transmission and the serological data supported his hypothesis ^12^. It was the first documented case of sexual transmission of ZIKV, but it garnered little attention from authorities, because no one could envision that ZIKV would cause such an epidemic eight years later. Dr. Foy suggested that sexual transmission is a matter of major concern, particularly now that there is growing evidence that ZIKV remains active in seminal fluid for six months ^13^ and localizes to and replicates very well in tissues of the urogenital tract ^14^ after both mosquito bite and sexual transmission. In short, the epidemic is unlikely to be controlled only by interrupting/terminating transmission by mosquito vectors.

### Genetically-based vector control strategies

Dr. Luciano Moreira discussed his laboratory findings indicating that a bacterium isolated from the southern house mosquito, *Cx. quinquefasciatus*, *Wolbachia* sp ( *w*Mel_Br strain), blocks transmission of ZIKV by the yellow fever mosquito *, Ae. aegypti*, and could be a useful tool for decreasing ZIVK transmission. Dr. Anthony James gave an overview of all genetic tools currently available in vector control. He explained that the strategy for population suppression is a genetic tool analogous to the development of an insecticide in the sense that the ultimate goal is to reduce or eliminate populations of vectors. Another strategy is population alteration (formerly known as population replacement) in which the ability of the mosquito to transmit a virus is changed, i.e., a gene engineered into the mosquito’s genome impacts the vector competence and, consequently, the virus does not replicate and is not transmitted. This is analogous to the *Wolbachia* strategy reported by Dr. Moreira. The two techniques have quite different approaches for an ultimate goal of reducing transmission of vector-borne diseases. The former is aimed at reducing mosquito populations and, consequently, reducing mosquito bites and transmission of viruses. On the other hand, with population alteration mosquitoes still bite, but no longer transmit the target virus. Lastly, Dr. James discussed a technique called gene drive, which allows a gene to move quickly into a population. This would have a long-term effect, so he emphasized the need for laboratory tests and contained facility experiments before full implementation in a vector control program. He concluded by suggesting that the best scenario is cases involving one single pathogen causing a disease and one single mosquito vector. Later, in the discussion he gave a specific example when answering questions from Dr. Thomas Scott, University of California-Davis. He suggested that these genetic tools might not be the best strategies for ZIKV given that at this point there seem to be multiple vectors not only at the species but also at the population level. The current genetic technologies would not be appropriately applied to such complex systems. He would not recommend any specific genetic strategy for ZIKV at this point. By contrast, he indicated that DENV might be a good candidate, particularly in areas where clearly there is only one vector species. Dr. James noted that in the *Aedes*-DENV case replacement might be the best alternative given that it is difficult to achieve population suppression of *Aedes* mosquitoes.

### Zika vectors

Dr. Fiona Hunter examined closely the phylogeny of ZIKV-related viruses and showed that ZIKV belongs to a transition point between classical *Culex-*associated and *Aedes*-associated viruses. Her analysis suggested that ZIKV belongs to a clade (supported 99%) of neurotropic viruses, including West Nile Virus (WNV) and Saint Louis Encephalitis (SLE) virus, which are typically transmitted by *Culex* mosquitoes. ZIKV does not belong to a clade of hemorrhagic viruses, such as DENV and yellow fever, which are typically transmitted by *Aedes* mosquitoes. In short, there is apparently a dichotomy between the mosquito vector species vis-à-vis the taxonomy of the virus per International Committee on Taxonomy of Viruses. She suggested that we should keep an open mind, because ZIKV might have a larger range of vectors. In her field studies in the Dominican Republic, she collected one ZIKV-infected *Culex* mosquito, but was not able to identify the mosquito to the species level because of the damage caused by her trapping system. Additionally, she reported preliminary data on vector competence of *Cx. pipiens* collected in Canada by RT-qPCR and plaque assays showing at least 2% ZIKV transmission with her ongoing analysis. In summary, her taxonomic analysis along with field and laboratory findings support her hypothesis for a wider range of ZIKV vectors.

Dr. Constância Ayres suggested that *Cx. quinquefasciatus* is being held to a different standard than *Ae. aegypti* with regard to ZIKV transmission and its potential role as a ZIKV vector has been overlooked. She started her presentation by stressing the textbook ^15^ criteria for incrimination of arthropods as vectors of humans and other animals. In particular, she emphasized that in multiple reported cases no data were available in the urban environment regarding criteria #3 and #4, i.e., “repeated demonstration that suspected vectors, collected under natural conditions, harbor the identifiable, infective stage of the pathogen” and “a biological association between clinical cases and infected mosquitoes in time and space,” respectively. For example, no infected mosquitoes were collected from Yap States and French Polynesia during the outbreaks. They studied vector competence under laboratory conditions and assumed that *Aedes* species were vectors, although they never fulfilled the above criteria. She stressed the fact that there are significant populations of *Cx. quinquefasciatus* in these locations, but they were not analyzed for ZIKV infection, and vector competence was not studied under laboratory conditions. Given this scenario, Dr. Ayres wrote a position paper ^16^ arguing the need to determine unambiguously whether *Cx. quinquefasciatus* is a ZIKV vector. Ever since, various studies have suggested that *Culex* species are not ZIKV vectors, whereas evidence from other studies (see below) strongly suggests that *Cx. quinquefasciatus* might be a significant vector. Of note, Aliota and collaborators ^17^ fed mosquitoes from laboratory colonies on mice previously infected with Asian lineage ZIKV strain PRVABC59. All samples from *Cx. pipiens* mosquitoes and all replicates were negative for ZIKV by plaque assay, 14 days post-infection (dpi). By contrast, at least one replicate for each, *Ae. aegypti and Ae. albopictus,* showed at least a 22% transmission rate. Huang *et al.* ^18^ studied infection and dissemination rates of 7 and 14 dpi and demonstrated that *Cx. pipiens* and *Cx. quinquefasciatus* (Vero Beach strain) were refractory to ZIKV, although they did not report a positive control. Fernandes ^19^ and collaborators achieved a remarkable feat. In a short period of time, they were able to capture *Cx. quinquefasciatus* from various suburbs in Rio de Janeiro and conduct vector competence studies under laboratory conditions using F1 generations. Although survival rate, yield of blood meals, and other parameters were not reported, their data showed zero transmission rates for *Cx. quinquefasciatus* at 7, 14, and 21 dpi. Their publication was widely highlighted in the press and social media, but a report from Guo *et al.* ^20^ that appeared the next day got absolutely no coverage. As discussed (see also below), this report provides clear evidence of infection, dissemination, replication in salivary glands and transmission to infant mice by *Cx. pipiens quinquefasciatus*. As it stands now, we should take Dr. Hunter’s advice and keep in mind that the jury is still out regarding ZIKV vectors. It might as well be that virus strains and/or mosquito populations account for the discrepancies. Dr. Ayres pointed out in her presentation that one should not forget the socioeconomic and ecologic factors, environment, and behavior of *Cx. quinquefasciatus* in Recife when considering the full range of vectors.

Dr. Duschinka Guedes presented solid evidence demonstrating that ZIKV was detected in midgut, salivary glands, and saliva of *Cx. quinquefasciatus* from Recife, Brazil, which were artificially infected with a strain of the ZIKV isolated from a local patient. In addition to conventional vector competence studies under laboratory conditions, Dr. Guedes showed that both *Cx. quinquefasciatus* and *Ae. aegypti* expectorated the virus into honey-soaked filter papers, 9–12 dpi. Additional evidence of replication in the salivary glands was demonstrated by transmission electron microscopy data. ZIKV-infected acinar cells showed signs of cytopathic disruptions, and mature ZIKV particles were clearly observed. Lastly, she reported that multiple pools of *Cx. quinquefasciatus* mosquitoes collected from urban areas with a high incidence of microcephaly in Recife were infected with ZIKV ^21^. Dr. Guedes’ presentation strongly suggests that in Recife, Brazil, both *Ae. aegypti* and *Cx. quinquefasciatus* are ZIKV vectors. Working independently in China, Dr. Tang-yan Zhao reached similar conclusions. Dr. Zhao performed conventional vector competence studies, which demonstrated ZIKV replication in midgut and salivary glands ^20^. Additionally, she placed 91-day-old infant mice in a cage with *Cx. quinquefasciatus* mosquitoes previously infected with ZIKV. The infant mice developed red blotches on the skin, and at 10 dpi eight of the nine mice had high titers of viral RNA in their brains. These two independent studies discussed at the Zika Symposium are complementary in nature and they both support the notion that *Cx. quinquefasciatus* might be a ZIKV vector. It is, therefore, advisable that vector management programs aimed at mitigating ZIKV transmission do not ignore *Cx. quinquefasciatus*, unless new and unambiguous evidence will show that a target population of the common mosquito is not a vector. For the time being, it is prudent to consider that vector competence may vary among different populations of the same species and/or the strains of the virus.

### Zika discussion

During the discussion at the end of the symposium, the forum was opened for questions and comments. “Is anyone looking for the virus in birds?” asked Dr. Scott Ritchie, James Cook University, Australia. This question captures the sentiment that both questions were thought provoking, and we still do not have all or many answers when it comes to ZIKV. Hopefully, we will be better prepared when convening in Finland for ICE 2020. Wouldn’t it be wonderful to report in Helsinki that mosquito vector populations have been reduced or eliminated, the Zika and other epidemics were contained, vaccines have been made available, and entomologists are ready to further improve the human condition by tackling other problems than the Zika epidemic?
