# Supplementary material for: Zika mosquito vectors: the jury is still out
Source: F1000Res. 2016 Oct 20;5:2546. [Version 1] doi: 10.12688/f1000research.9839.1 (PMC5105876; doi:10.12688/f1000research.9839.1)
Supplement: Peer review history

# Peer review: Referee response for version 1

**DOI:** 10.5256/f1000research.10608.r17269

**Published:** 2016-11-09

**Authors:** Jeffery K. Tomberlin

**Affiliations:** 1 Department of Entomology, Texas A&M University, College Station, TX, USA

The title and abstract are very appropriate as they give the reader a quick overview of the subject matter discussed as related to Zika. This opinion piece provides a brief background of the International Congress of Entomology with an emphasis on a symposium exploring what is, and not, known about Zika. The article summarizes presentations given by a number of globally recognized researchers in the field of medical entomology and disease ecology. The article does an excellent job providing an overview of where society, and research, stands today with regards to Zika and its potential spread globally. However, as they discuss, additional research is needed to fully understand the ecology of the pathogen and vector capacity as related to disease manifestations and frequency of occurrence. I would like to stress the outbreak of Zika is a demonstration of the need to continue exploring the mechanisms regulating mosquito, and other arthropod vectors, attraction to, and blood-feeding on, hosts as the development of methods to disrupt such interactions could prove vital in protecting human populations and their associated livestock globally.

I have read this submission. I believe that I have an appropriate level of expertise to confirm that it is of an acceptable scientific standard.

# Peer review: Referee response for version 1

**DOI:** 10.5256/f1000research.10608.r17437

**Published:** 2016-11-09

**Authors:** Andre Freire Furtado

**Affiliations:** 1 Centro de Pesquisas Aggeu Magalhães , Recife, Brazil

In this opinion article, Dr. Walter S. Leal presents not only the discrepant results in the literature concerning the vector or vectors mosquitoes of Zika (ZIKV) – (Aedes, Culex), but gives an up-to-date knowledge about the history of ZIKV infection and its consequence on the human health.

 Dr. Leal clearly describes all the steps since the ZIKV isolation from a febrile monkey of Uganda forest, the isolation of ZIKV from human in 1954 and presents the results (opinions) of different groups of research, concerning the responsible vector of ZIKV transmission as well as the different concerns to explain the discrepancies.The paper also brings information about ZIKV epidemics in the world, the sexual transmission, and a very helpful discussion on the genetically based vector control strategies. A disease considered as a mild infection in the beginning is now well established as causing microcephaly through congenital infection. This teratogenic effect led the World Health Organization (WHO) to declare ZIKV an international public health emergency.

 I read this submission and my opinion is that it is worth to be indexed considering its high standard.

I have read this submission. I believe that I have an appropriate level of expertise to confirm that it is of an acceptable scientific standard.

# Peer review: Referee response for version 1

**DOI:** 10.5256/f1000research.10608.r17268

**Published:** 2016-11-08

**Authors:** Mario Alberto Cardoso da Silva-Neto

**Affiliations:** 1 Instituto de Bioquímica Medica Leopoldo de Meis, Universidade Federal do Rio de Janeiro, Rio de Janeiro, Brazil

I have read the MS "Zika mosquito vectors: the jury is still out" by Walter S. Leal Department of Molecular and Cellular Biology, University of California-Davis, Davis, USA. This MS nicely describes the scientific and academic atmosphere and major findings of International Congress of Entomology (ICE 2016) held in Orlando, Florida (September 25-30, 2016). The MS goes far beyond the simple description of the common routine in Meetings such like this. In fact due to our major and now global concern on arboviroses the MS provides a precise account of the most relevant issues discussed on this meeting. Also, it poses an ultimate perspective that should be urgently followed regarding the discussion of the true vectors of Zika. Such relevant points must be immediately addressed concerning the final definition of the molecular and genetic strategies to be used for its control which were also discussed in the meeting and reported in the present MS. I believe the scientific content and the present format of this MS are suitable for indexing.

I have read this submission. I believe that I have an appropriate level of expertise to confirm that it is of an acceptable scientific standard.

# Peer review: Referee response for version 1

**DOI:** 10.5256/f1000research.10608.r17135

**Published:** 2016-11-07

**Authors:** Coby Schal

**Affiliations:** 1 Department of Entomology, W.M. Keck Center for Behavioral Biology, North Carolina State University (NCSU), Raleigh, NC, USA

In summarizing a timely and well-attended symposium at the ICE2016 conference, Leal’s opinion article also masterfully encapsulates the history of the Zika epidemic and glaring gaps in our understanding of Zika virus transmission. While still in its early phase, the Zika outbreak is a relatively “minor” public health event on a global scale, compared to other infectious and arboviral diseases, such as Malaria. But because of its astonishingly rapid emergence, especially in Brazil, its unusual damaging effect on neurological development of the human neonatal brain, its spectacular evolution since it was first isolated, and its sexual transmission and stealthy existence in seminal fluid in humans, this virus has “gone viral”, especially during the 2016 summer Olympics in Rio de Janeiro. Leal’s summary highlights important needs in the research agenda to combat Zika. All public health interventions rally around “source/vector reduction”, but the full array of mosquito species that are competent to vector the Zika virus remains to be determined. Recent research has added genetic approaches to the tool-box for vector management, but it will be years before these powerful options can be broadly implemented.

 A couple of corrections and editorial points:

1. Consider replacing “conquering” with “spreading into” in the sentence: “ZIKV was silently making headway out of the Zika forest (Entebbe, Uganda), hitchhiking on humans, and conquering new habitats” (p.2).
2. Change “91-” to “nine one-” in the sentence: “Additionally, she placed 91-day-old infant mice in a cage” (p.5).

I have read this submission. I believe that I have an appropriate level of expertise to confirm that it is of an acceptable scientific standard.

# Peer review: Referee response for version 1

**DOI:** 10.5256/f1000research.10608.r17310

**Published:** 2016-10-31

**Authors:** Pedro Lagerblad de Oliveira

**Affiliations:** 1 Laboratório de Bioquímica de Artrópodes Hematófagos, Instituto de Bioquímica Médica Leopoldo de Meis, Programa de Biologia Molecular e Biotecnologia, Universidade Federal do Rio de Janeiro, Rio de Janeiro, Brazil

This opinion article is essentially a meeting report, where most of the speakers showed data indicating that Culex mosquitoes could be vectors of Zika. However, as pointed by Dr Leal, several other groups published independent reports with negative results for Culex infection with Zika. While the major claim of this paper (that the vector competence of Culex for Zika is still an open question) seems a fair statement, reading of the published papers do not provide an immediate explanation for the discrepant results from different groups. Of course, although experimental errors can not be excluded, data can be reconciled as differences in virus and or mosquito strains (or even symbiont microbiota) can explain the distinct findings from each group. If Zika transmission was merely an academic question, I would say that time would solve the question. However, this is a major threat for global public health, and transmission mediated by one or two species calls for very distinct control strategies. Therefore, I would add to this report that there this debate needs urgently to evolve in the experimental field, ideally as collaborative research, but at least with exchange of virus and mosquitoes strains, and rigorous comparisons of the methodology.

I have read this submission. I believe that I have an appropriate level of expertise to confirm that it is of an acceptable scientific standard.
